# Hippocampal Subfield Volumetry and Navigation in Congenital Blindness

**DOI:** 10.1002/hipo.70115

**Published:** 2026-08-04

**Authors:** Daniel‐Robert Chebat, Fabien C. Schneider, Ron Kupers, Maurice Ptito

**Affiliations:** ^1^ The Department of Psychology Ariel University Ariel Israel; ^2^ Université Jean Monnet Saint‐Etienne, CHU Saint‐Etienne, Unité de recherche TAPE, EA 7423 Saint‐Etienne France; ^3^ École d’Optométrie, Université de Montréal Québec Canada; ^4^ Institute of Neuroscience (IoNS), Faculté de Médecine, Université Catholique de Louvain (UCL) Brussels Belgium; ^5^ Department of Applied Mathematics and Computer Science, Visual Computing Danish Technical University Lyngby Denmark

**Keywords:** congenital blindness, hippocampal subfields, learning, MRI volumetry, neuroplasticity, obstacle detection and avoidance, sensory substitution, spatial navigation, structure–behavior relationships

## Abstract

The hippocampus is essential for efficient navigation. Although lack of visual experience from birth induces volumetric and structural modifications to the hippocampus, tactile and auditory navigation remain partially preserved in congenitally blind (CB) individuals. Structural studies in this population have relied on global volume or tail–body–head segmentations, leaving the contributions of individual hippocampal subfields to navigation unresolved. We investigated hippocampal subfield volumes and their association with navigational learning in CB adults compared to sighted controls. Structural MRI data were analyzed using probabilistic cytoarchitectonic ROIs to quantify volumes of Cornu Ammonis (CA), Fascia Dentata (FD), Subiculum (SUB), and the Hippocampal–Amygdaloid Transition Area (HATA). Participants performed obstacle detection and avoidance tasks with a tactile sensory substitution device in a real‐world obstacle course. CB participants showed significant volume reductions in left CA and FD compared to sighted controls after total brain volume correction. Obstacle detection learning was associated with bilateral CA and SUB volumes in sighted controls, whereas in CB participants it was selectively associated with right CA and right HATA volumes. Obstacle avoidance learning showed no robust hippocampal associations in either group. Our results indicate that hippocampal subfield–navigation associations are selective and component‐dependent in the absence of vision, rather than reflecting uniform hippocampal involvement.

**Abbreviations**
CA: Cornu Ammonis
CB: Congenitally Blind
FD: Fascia Dentata (Dentate Gyrus)
HATA: Hippocampal–Amygdaloid Transition Area
LoA: Learning of Avoidance
LoD: Learning of Detection
MRI: Magnetic Resonance Imaging
SC: Sighted Controls
SUB: Subiculum
TBV: Total Brain Volume
TDU: Tongue Display Unit

## 1 Introduction

Congenital blindness (CB), the complete absence of visual input from birth, provides a model to examine how visual experience modulates hippocampal organization and its relationship to navigation and navigational learning. Neural activity driven by early sensory experience influences both the development and later refinement of neural circuits (Pan and Monje 2020), and early sensory deprivation can lead to widespread reorganization of brain structure and function across many distributed neural systems (Ptito et al. 2008; Kupers and Ptito 2014). The hippocampus plays a central role in spatial cognition, supporting navigation, memory, learning and cognitive map formation (O'Keefe and Nadel 1978; Burgess et al. 2002; Moser et al. 2017). Furthermore, its organization is sensitive to experience‐dependent structural changes (Maguire et al. 2006).

Navigation can be defined as the selection of a trajectory between the current location and a target location (Montello 2005; Wolbers and Hegarty 2010). Behavioral studies have shown that navigation is not a unitary process but can be decomposed into dissociable components that rely on partially distinct cognitive and neural mechanisms (Byrne et al. 2007; Wolbers and Wiener 2014). Despite the absence of visual input, CB individuals often demonstrate efficient navigation when relying on route‐based and other egocentric strategies supported by non‐visual cues (Tinti et al. 2006; Chebat et al. 2011, 2015). Neuroimaging evidence further indicates that egocentric navigation recruits human visual area V6 even in the absence of visual experience (Aggius‐Vella et al. 2023). In contrast, performance is less reliable when tasks require allocentric or survey representations, or flexible switching between egocentric and allocentric reference frames (Iachini et al. 2014; Ruggiero et al. 2018; Pasqualotto et al. 2013). This distinction is particularly relevant for sensory‐substitution‐based navigation, which requires the integration of object identification, spatial localization, and action guidance—corresponding to “what,” “where,” and “how” processing (Proulx et al. 2018). This framework aligns with the present distinction between obstacle detection and obstacle avoidance, providing a psychological basis for examining whether hippocampal subfields contribute differentially to distinct components of non‐visual navigation learning. Neuroimaging studies have shown that the hippocampus (Gagnon et al. 2012), and parahippocampus (Kupers et al. 2010) remain functionally engaged during tactile maze navigation in CB, and meta‐analytic evidence supports this claim (Bleau et al. 2022; Schinazi et al. 2016). However, hippocampal engagement alone does not specify which aspects of navigation depend on hippocampal subfields. Such contributions are more likely to be expressed in experience‐dependent learning than in static task performance, consistent with real‐world evidence that hippocampal anatomy predicts rapid acquisition and flexible use of cognitive‐map knowledge (Schinazi et al. 2013).

Despite functional evidence of hippocampal engagement, structural findings in CB remain inconsistent. Most studies have relied on global hippocampal volumetry or anterior–posterior (tail–body–head; TBH) segmentation (Chebat et al. 2007; Fortin et al. 2008; Leporé et al. 2009; Paré et al. 2023), reporting divergent patterns. These include selective posterior reductions (Chebat et al. 2007), larger hippocampal volumes associated with superior navigation performance (Fortin et al. 2008), and right‐lateralized anterior–posterior shape dissociations (Leporé et al. 2009). These reports focused on global and longitudinal‐axis hippocampal segmentation approaches (e.g., Pruessner et al. 2000; Maguire et al. 2006), rather than cytoarchitectonic subfield analyses. While TBH segmentation provides a convenient anatomical framework, it does not map directly onto the hippocampus's cytoarchitectonic organization and may obscure subfield‐specific structure–behavior relationships (Poppenk et al. 2013; Strange et al. 2014; Collin et al. 2015).

**FIGURE 1:**
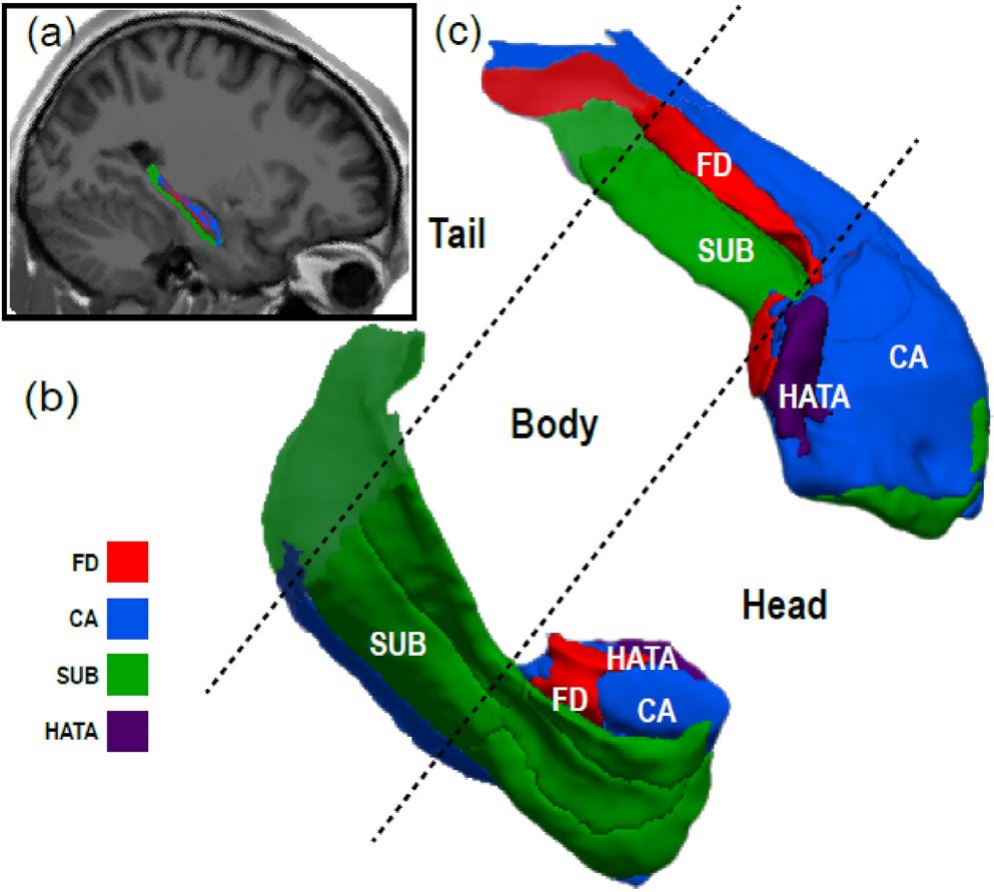
Tail–body–head (TBH) segmentation versus cytoarchitectonic subfields of the hippocampus. (a) Sagittal structural MRI illustrating the anatomical location of the hippocampus and regions of interest. (b) Three‐dimensional rendering of the left hippocampus shown in a dorsal view, with cytoarchitectonically defined subfields color‐coded and displayed relative to the traditional tail–body–head (TBH) division. (c) Ventral view of the same hippocampal segmentation, illustrating the spatial extent and continuity of subfields along the anterior–posterior axis. Dashed lines indicate conventional TBH boundaries. In the analysis, subfield volumetry did include posterior hippocampal voxels defined by the probabilistic atlas.

Although these findings highlight experience‐dependent hippocampal plasticity in CB, prior structural studies have relied on global hippocampal volumetry or traditional tail–body–head (TBH) segmentation along the longitudinal axis (Figure 1A) (Chebat et al. 2007; Fortin et al. 2008; Leporé et al. 2009). More specifically, subfield‐level volumetry—when evaluated relative to global brain size and internal hippocampal organization—enables dissociation between hippocampus‐specific volumetric differences and potential changes in internal hippocampal composition—distinctions that cannot be resolved using global hippocampal volume or longitudinal‐axis (TBH/anterior–posterior) subdivisions alone. At the functional level, resting‐state fMRI studies have reported altered hippocampal network organization in blindness, including changes in functional coupling along the hippocampal longitudinal axis in both congenitally and late blind individuals (Ma et al. 2017). At the structural level, surface‐based morphometric analyses have revealed spatially heterogeneous structural alterations of the hippocampal formation in both congenitally and late blind individuals (Pan et al. 2021). Consistent with this TBH delimitation, studies in sighted participants have shown that individual differences in hippocampal size predict the rapid acquisition of a cognitive map (Schinazi et al. 2013).

In contrast, the hippocampal formation comprises distinct subfields with specialized computational roles (Figure 1B). Cornu Ammonis (CA) regions support allocentric spatial coding and place‐based representations (O'Keefe and Nadel 1978; Burgess et al. 2002; Moser et al. 2017; Danjo 2020); the dentate gyrus (Fascia Dentata, FD) is critical for pattern separation and spatial discrimination (Leutgeb et al. 2007; Yassa and Stark 2011); the subiculum (SUB) serves as a major output node linking the hippocampus to cortical navigation networks (Witter and Amaral 2004; O'Mara 2005); and the hippocampal–amygdaloid transition area (HATA) integrates mnemonic and multisensory information at the interface of hippocampus and amygdala (Pitkänen et al. 2000; Amunts et al. 2005). Importantly, these subfields span across TBH divisions, which limits the ability to use TBH‐based segmentation for capturing functionally meaningful organization.

To date, no study has examined whether cytoarchitectonically defined hippocampal subfields show distinct structure–behavior relationships with dissociable navigation components in CB, nor whether these relationships differ from those observed in sighted controls (SC). In the present study, we reanalyzed structural MRI and behavioral navigation data acquired from the same cohort of CB and sighted control participants. The structural MRI acquisition and behavioral navigation procedures have been described previously (Chebat et al. 2020a). Using an atlas‐based, cytoarchitectonic subfield framework, we quantified volumes of CA, FD, SUB, and HATA, and examined their relationships with learning during a real‐world obstacle‐course navigation task performed with a sensory substitution device, dissociating obstacle detection from avoidance. We hypothesized that CB would show selective, rather than uniform, differences in hippocampal subfield contributions to navigation learning, and that structure–behavior relationships between hippocampal subfields and specific components of navigation would differ between CB and SC groups. Specifically, we expected hippocampal subfield volumes to be more strongly associated with learning‐related changes in obstacle detection and avoidance than with static task performance, with group‐specific patterns reflecting differences in navigational strategies and sensory experience between CB and SC participants.

## 2 Materials and Methods

### 2.1 Participants

Twelve CB adults (7 males; mean age = 35 years, SD = 10.92, range = 20–54) and 21 SC participants (13 males; mean age = 33 years, SD = 11.87, range = 22–68) were included in the study (Table 1). Age did not differ significantly between groups. All CB participants had no functional visual experience. Participants provided written informed consent prior to participation. The study was approved by the Ethics Committee of the Université de Montréal and conducted in accordance with the Declaration of Helsinki. Portions of the MRI (Chebat et al. 2020a) and behavioral datasets have been reported previously (Chebat et al. 2011), and the present study reanalyzed these data to address the relationship between hippocampal subfields and navigational skills.

**TABLE 1:** Characteristics of the congenitally blind participants.

Participant	Sex	Age	Onset	Cause of blindness	Light perception
1	M	36	Birth	Retinopathy of prematurity	None
2	M	48	2 months	Electrocution	None
3	F	49	Birth	Retinopathy of prematurity	None
4	M	41	Birth	Retinitis pigmentosa	None
5	F	24	Birth	Detached retina	None
6	M	38	Birth	Retinopathy of prematurity	None
7	F	31	Birth	Glaucoma, aniridia	None
8	M	20	Birth	Leber's congenital amaurosis	None
9	M	34	Birth	Glaucoma	None
10	M	23	Birth	Congenital cataract	None
11	F	29	Birth	Retinoblastoma	None
12	F	54	Birth	Retinopathy of prematurity	None

### 2.2 MRI Acquisition

MRI data were acquired in Montreal, Canada, on a 1.5 T Siemens Vision scanner (Siemens Medical Systems, Erlangen, Germany), as described previously (Ptito et al. 2008). T1‐weighted images were collected using a fast spoiled gradient‐echo sequence (TI/TR/TE = 450/106/42 ms, flip angle = 20°), at a spatial resolution of 0.94 × 0.94 × 1 mm.

### 2.3 Image Preprocessing and Hippocampal Subfield Volumetry

Structural MRI data were preprocessed using a voxel‐based morphometry pipeline, following procedures described in detail elsewhere (Ptito et al. 2008; Chebat et al. 2020a). For each participant, averaged gray matter volumes (ROI‐wise gray matter volume estimates) were extracted within predefined probabilistic cytoarchitectonic ROIs from the SPM Anatomy Toolbox (Amunts et al. 2005; Eickhoff et al. 2005). This approach enables anatomically grounded subfield‐level analyses beyond global or longitudinal‐axis hippocampal segmentations used in earlier work. Analyses focused on the CA, FD, SUB and HATA, hippocampal subfields relevant to spatial cognition. In the present study, we employed probabilistic cytoarchitectonic ROIs from the SPM Anatomy Toolbox, which provide anatomically validated subfield definitions aligned with the spatial resolution and acquisition characteristics of the dataset. This approach enables conservative and interpretable subfield‐level structure–behavior analyses within the hippocampus. In line with classical anatomical definitions, we operationalized hippocampal volume as the hippocampus proper: Cornu Ammonis, dentate gyrus (fascia dentata), and subiculum, excluding adjacent transitional regions such as the hippocampal–amygdaloid transition area (HATA) and entorhinal cortex (Witter and Amaral 2004; Strange et al. 2014). The volume of the hippocampus proper was calculated as the sum of CA, FD, and SUB volumes within each hemisphere (excluding HATA), consistent with our prior reports; HATA and EC were analyzed and reported separately. To dissociate hippocampus‐specific effects, and potential shifts in internal hippocampal composition, subfield volumes were examined at two complementary levels: (i) subfield volumes corrected for total brain volume (TBV) to isolate hippocampus‐specific effects beyond global brain size, and (ii) proportional subfield volumes normalized to ipsilateral total hippocampus proper volume (subfield/hippocampus), to assess relative subfield contributions independent of overall hippocampal size. TBV‐adjusted analyses were implemented as ANCOVAs with TBV entered as a covariate. Proportional analyses used subfield/ipsilateral total hippocampus proper volume ratios and were tested on these ratios. Group differences in proportional subfield organization (Table 4) were assessed using independent‐samples *t*‐tests with unequal variances (Welch's correction; two‐tailed).

### 2.4 Navigation Task and Learning

**FIGURE 2:**
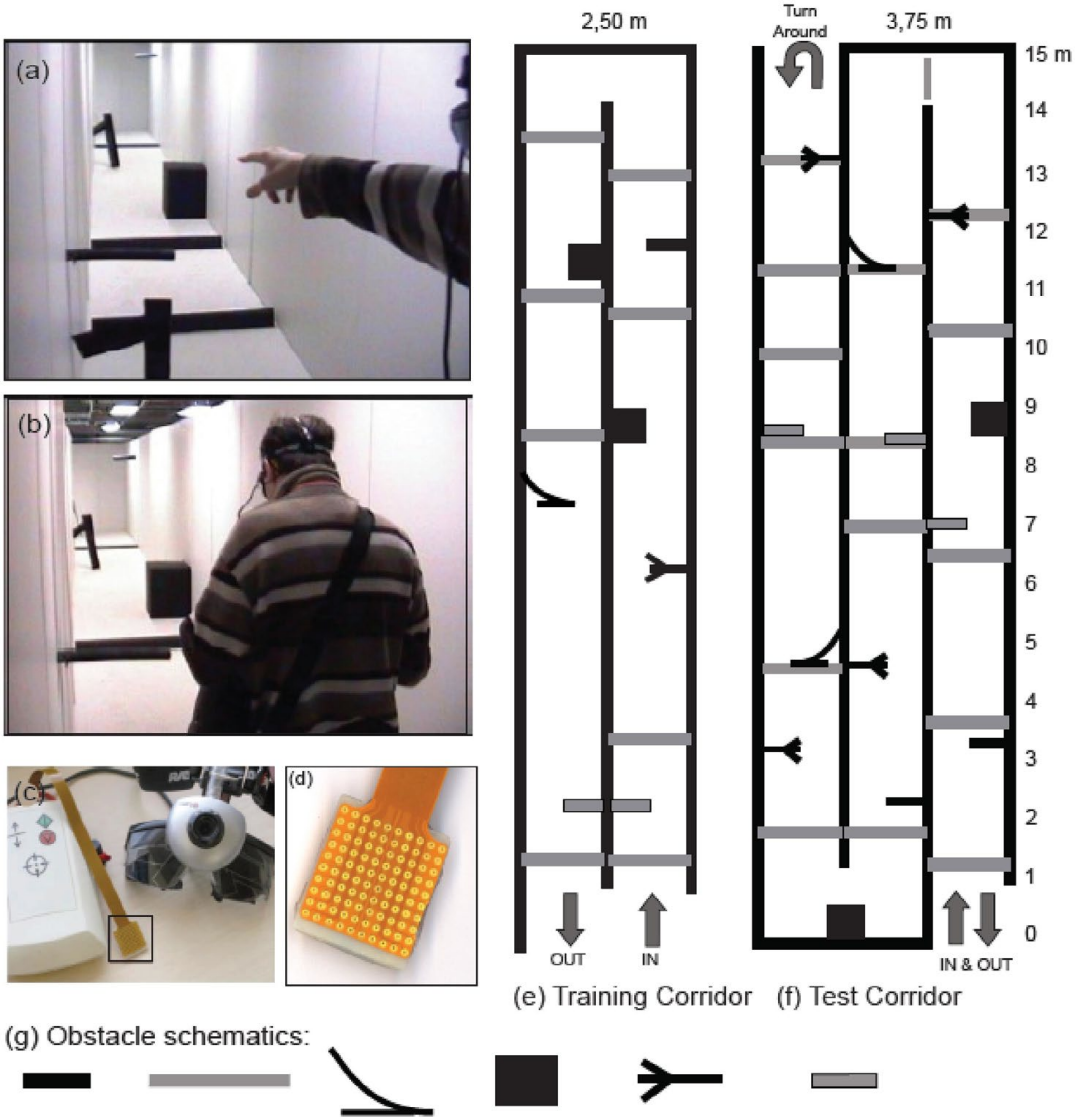
Experimental tasks and sensory substitution apparatus. (a) Pointing task. A participant indicates the perceived direction of a target while standing in the experimental corridor. (b) Navigation task. A participant navigates through an obstacle course via sensory‐substitution feedback. (c) The Tongue Display Unit (TDU) system used in the experiment, including the camera and processing unit connected to the intra‐oral electrode array. (d) Close‐up view of the tongue array 10 × 10 matrix of electrodes delivering electrotactile stimulation to the tongue. (e) Schematic layout of the training corridor, consisting of two parallel hallways with multiple obstacle configurations. (f) Schematic layout of the test corridor, consisting of three parallel hallways and a distinct obstacle arrangement relative to training. Arrows indicate traversal direction and turning points. (g) Obstacle schematics illustrate the different obstacle geometries used in the task, including ground‐level, elevated, and volumetric obstacles positioned at varying heights and locations along the corridor. Schematics are not drawn to scale.

Navigation performance and learning were assessed using a real‐world obstacle course navigation task (Figure 2a,b) performed with a visuo‐tactile sensory substitution device, the Tongue Display Unit (TDU) (Figure 2c,d), which converts visual information into electro‐tactile stimulation applied to the tongue (Bach‐y‐Rita et al. 1998). The experimental setup (Figure 2e–g), apparatus, and general training procedures have been described in detail previously (Chebat et al. 2020a). All participants, regardless of visual status, completed the navigation paradigm under blindfolded conditions, had no prior experience with the TDU, and underwent the same standardized familiarization and training procedure before testing. Participants then navigated a life‐size obstacle course containing ecologically valid objects of varying shapes and sizes while equipped with the TDU.

Participants performed a navigation task requiring obstacle detection and avoidance while using a tongue‐based sensory substitution device (Figure 2c) connected to a tongue electrode array (Figure 2d). During each run, they pointed toward detected obstacles and then negotiated a collision‐free path around or over them (Figure 2a,b). The task included a training phase in a two‐hallway corridor (Figure 2e) and a test phase in a three‐hallway corridor (Figure 2f). Obstacle types are illustrated schematically in Figure 2g and were designed to resemble common environmental barriers. Participants completed four runs in the training corridors and then walked through the testing phase corridors. Performance was quantified as the percentage of correctly detected and avoided obstacles. Detection was scored as correct when an obstacle was identified prior to contact, and avoidance when it was passed without contact. The experimenter followed behind participants at a short distance to ensure safety. The task was structured to assess learning between the training phase and testing phases. Learning was operationalized as the change in performance between the training and the test phase (Chebat et al. 2020b). Separate indices were computed for learning of detection (LoD) and learning of avoidance (LoA), following previously established procedures (Chebat et al. 2020a). *LoD and LoA* served as the primary behavioral outcome variables in the structure–behavior analyses.

### 2.5 Statistical Analysis

All statistical analyses were conducted using IBM SPSS Statistics (version 20; IBM Corp., Armonk, NY, USA). Data normality was assessed using the Shapiro–Wilk test. One congenitally blind participant had missing behavioral data and was excluded from paired learning analyses (CB *n* = 11). Group differences in hippocampal subfield volumes were examined using analyses of covariance (ANCOVAs), with total brain volume included as a covariate where appropriate. Percent differences between groups were calculated as ((CB − SC)/SC) × 100. Structure–behavior relationships were assessed using Pearson correlations between hippocampal subfield volumes and navigation performance, indexed by *detection learning* and *avoidance learning*. Statistical significance was set at *p* < 0.05 (two‐tailed) for all analyses. Correlation analyses were corrected for multiple comparisons using the Benjamini–Hochberg false discovery rate procedure (*q* = 0.05; Benjamini and Hochberg 1995). Consistent with our a priori, hemisphere‐specific hypotheses, FDR correction was applied separately within each group × behavioral domain × hemisphere family, correcting across the four hippocampal subfields (CA, FD, SUB, HATA) tested within each hemisphere. All analyses were hypothesis‐driven and restricted to predefined regions of interest within the hippocampus (CA, FD, SUB, HATA), selected based on established roles in spatial cognition and navigation (O'Keefe and Nadel 1978; Burgess et al. 2002; Poppenk et al. 2013; Strange et al. 2014; Collin et al. 2015). We therefore defined two behavioral indices, LoD and LoA, reflecting distinct cognitive components.

## 3 Results

Results are presented for behavioral performance, hippocampal subfield volumes, and structure–behavior relationships.

### 3.1 Behavioral Results

Descriptive statistics for training and test performance, as well as learning of detection (LoD) and avoidance (LoA), are summarized in Table 2. Both CB and SC participants improved from training to test phases for detection and avoidance, confirming learning across repeated exposure to the task. There were no significant group differences in LoD or LoA, indicating comparable behavioral improvement across groups. These learning indices served as the primary behavioral measures in all subsequent structure–behavior analyses.

**TABLE 2:** Training, test, and learning scores for obstacle detection and avoidance.

Measure	Group	Training (Mean ± SE)	Test (Mean ± SE)	Learning (Mean ± SE)	Paired *t*‐test (two‐tailed)
Detection	SC	53.48 ± 4.15	68.57 ± 5.23	15.10 ± 2.12	*t*(20) = 7.06, *p* < 0.001
	CB	62.18 ± 5.72	71.64 ± 4.56	9.45 ± 2.63	*t*(10) = 3.60, *p* = 0.005
Avoidance	SC	45.38 ± 3.74	59.86 ± 3.99	14.48 ± 1.92	*t*(20) = 7.41, *p* < 0.001
	CB	33.00 ± 3.83	54.09 ± 6.67	21.09 ± 5.13	*t*(10) = 4.11, *p* = 0.002

### 3.2 Hippocampal Subfield Volumetry

**FIGURE 3:**
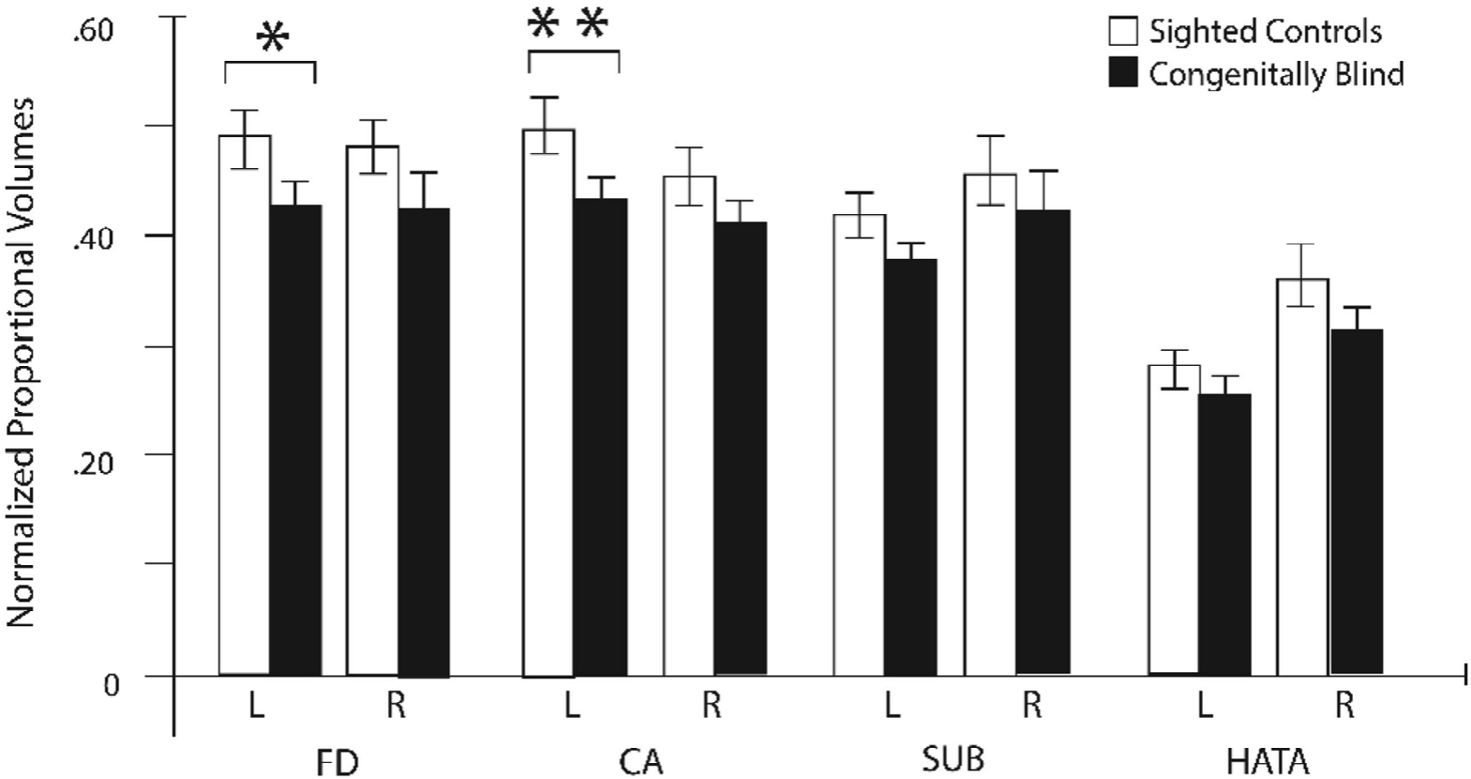
Group differences in hippocampal subfield volumes after total brain volume (TBV) correction. Bar graphs show mean TBV‐adjusted gray‐matter volumes (± SD) for each hippocampal subfield included in the analysis: Cornu Ammonis (CA), Fascia Dentata (FD), Subiculum (SUB), and the Hippocampal–Amygdaloid Transition Area (HATA), shown separately for congenitally blind (CB) participants and sighted controls (SC). Asterisks indicate statistically significant group differences after TBV correction (* *p* < 0.05, **p < 0.01; see Table 3).

After TBV correction, group differences remained significant for left CA (*p* = 0.008) and left FD (*p* = 0.040), whereas differences in right CA, FD, SUB, and HATA were no longer significant (Table 3). After TBV correction, total hippocampus proper volume (CA + FD + SUB) also remained significantly smaller in the CB group compared to SC (*p* = 0.019; Table 3). The TBV‐adjusted group differences in hippocampal subfield volumes are illustrated in Figure 3. To determine whether these volumetric differences reflect global hippocampal scaling or selective internal reorganization, we next examined proportional subfield organization by normalizing each subfield volume to the ipsilateral hippocampal formation volume.

**TABLE 3:** TBV‐adjusted hippocampal subfield gray‐matter volumes by group.

Region	CB (Mean ± SD)	SC (Mean ± SD)	% Difference	ANCOVA (*p*, TBV‐corrected)
Hipp (FD) L	0.424 ± 0.056	0.488 ± 0.054	−13.2%	*p* = 0.040*
Hipp (FD) R	0.424 ± 0.056	0.480 ± 0.048	−11.7%	*p* = 0.074
Hipp (SUB) L	0.376 ± 0.036	0.417 ± 0.042	−9.8%	*p* = 0.132
Hipp (SUB) R	0.418 ± 0.038	0.460 ± 0.040	−9.1%	*p* = 0.093
Hipp (CA) L	0.429 ± 0.050	0.498 ± 0.052	−13.9%	*p* = 0.008**
Hipp (CA) R	0.405 ± 0.046	0.455 ± 0.052	−11.0%	*p* = 0.159
Hipp (HATA) L	0.254 ± 0.044	0.280 ± 0.040	−9.2%	*p* = 0.772
Hipp (HATA) R	0.307 ± 0.042	0.357 ± 0.057	−14.2%	*p* = 0.186
(Hipp Proper) L	1.229 ± 0.134	1.404 ± 0.142	−12.5%	*p* = 0.029*
(Hipp Proper) R	1.247 ± 0.134	1.395 ± 0.136	−10.6%	*p* = 0.071
(Hipp Proper) Bi	2.476 ± 0.259	2.799 ± 0.270	−11.5%	*p* = 0.019*

*Note:* Values represent gray‐matter volumes extracted from probabilistic cytoarchitectonic ROIs and adjusted for total brain volume (TBV) using ANCOVA. Negative percent differences indicate lower gray‐matter volumes in congenitally blind (CB) participants relative to sighted controls (SC). “Total” hippocampal volume corresponds to CA + FD + SUB (excluding HATA); HATA is reported as a separate ROI. Bold values indicate statistically significant group differences. *p < 0.05; **p < 0.01.

### 3.3 Proportional Subfield Organization

Subfield volumes were normalized by dividing each subfield volume by the ipsilateral hippocampal formation volume to assess potential differences in internal hippocampal composition. Because these values are expressed as proportions, group differences are reported both as percentage‐point differences (Δ, CB − SC) and as relative percent change (Table 4). This analysis revealed a significant proportional reduction in left CA in the CB group relative to SC (*p* = 0.020). No other subfield, including FD, SUB, or HATA in either hemisphere, showed significant proportional differences between groups (all *p*'s > 0.10).

**TABLE 4:** Proportional hippocampal subfield volumes by group.

Subfield	CB Mean (SD)	SC Mean (SD)	Δ (pp)	Relative difference (%)	*p*
FD‐L	21.83 (1.11)	22.48 (1.10)	−0.65	−2.9%	*p* = 0.115
FD‐R	20.38 (1.07)	20.78 (0.83)	−0.40	−2.0%	*p* = 0.269
SUB‐L	19.44 (0.72)	19.22 (0.66)	+0.22	+1.1%	*p* = 0.387
SUB‐R	20.17 (0.65)	19.94 (0.54)	+0.23	+1.1%	*p* = 0.319
CA‐L	22.15 (0.98)	22.96 (0.60)	−0.81	−3.5%	*p* = 0.020*
CA‐R	19.52 (0.55)	19.69 (0.53)	−0.17	−0.9%	*p* = 0.389
HATA‐L	13.08 (1.37)	12.90 (1.36)	+0.18	+1.4%	*p* = 0.721
HATA‐R	14.76 (1.10)	15.40 (1.25)	−0.64	−4.2%	*p* = 0.136

*Note:* Values are proportional subfield volumes normalized to ipsilateral hippocampal formation volume and expressed as percentages. Δ (pp) denotes the difference in percentage points (CB − SC). Relative difference (%) was computed as (CB − SC)/SC × 100. Group differences were tested using independent‐samples *t*‐tests with unequal variances (Welch's correction; two‐tailed). Positive values indicate proportionally greater representation in congenitally blind (CB) participants relative to sighted controls (SC). Bold values indicate statistically significant group differences. *p < 0.05.

### 3.4 Structure–Behavior Relationships

**FIGURE 4:**
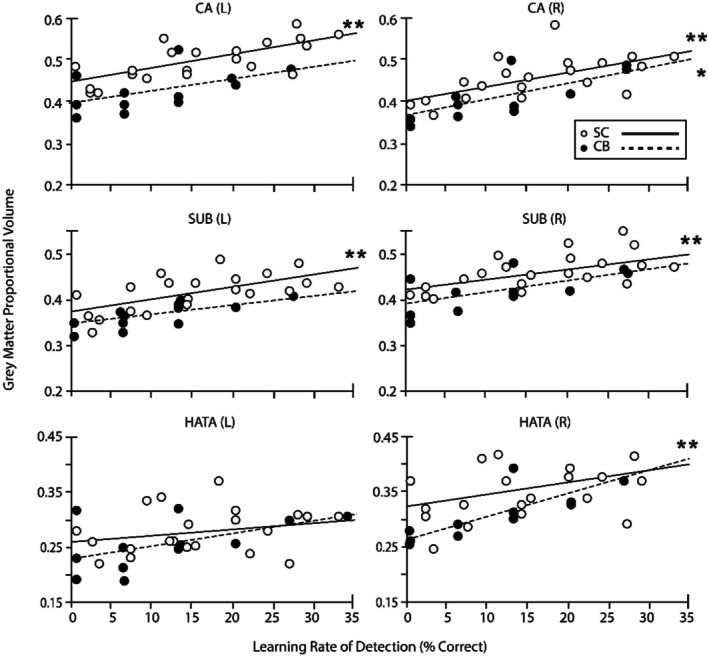
Hippocampal subfield volumes and LoD. Scatterplots show associations between hippocampal subfield gray‐matter volumes and the learning of detection (LoD), computed as the change in obstacle detection performance from training to test. Congenitally blind participants (CB; black filled circles, dashed regression lines) and sighted controls (SC; white circles, solid regression lines) are plotted within the same panels. Rows correspond to subfields (top: Cornu Ammonis [CA]; middle: Subiculum [SUB]; bottom: Hippocampal–amygdaloid transition area [HATA]); columns correspond to hemispheres (left, right). Pearson's *r* values (with exact *p*‐values and FDR‐corrected significance) are reported in Table 5.

To examine how hippocampal subfields relate to learning, Pearson correlations were computed between subfield volumes and LoD and LoA, separately for each group (Table 5). As a complementary analysis, we also tested correlations between hippocampal subfield volumes and raw detection and avoidance performance during the training and test phases (analyzed separately). These associations were weak and inconsistent, with no significant correlations in the CB group, and only nominal effects in SC that did not remain significant after correction for multiple comparisons. Total hippocampus proper volume (CA + FD + SUB; excluding HATA) was also correlated with LoD (SC: *r* = 0.59, *p* = 0.005; CB: *r* = 0.62, *p* = 0.033), but not with LoA (ps ≥ 0.057). False discovery rate correction (Benjamini–Hochberg, *q* = 0.05) was applied within each group × behavioral domain × hemisphere family, correcting across the four hippocampal subfields tested within each hemisphere. Exact *p*‐values and FDR‐corrected significance are reported in Table 5. Visual inspection of the scatterplots (Figure 4) confirmed that these associations were not driven by single extreme outliers.

**TABLE 5:** Correlations between hippocampal subfield volumes and obstacle‐specific learning.

ROI	LoD CB *r* (*p*)	LoD SC *r* (*p*)	LoA CB *r* (*p*)	LoA SC *r* (*p*)
CA‐L	0.53 (0.093)	**0.62 (0.003)**	0.24 (0.486)	0.22 (0.348)
CA‐R	**0.68 (0.020)**	**0.61 (0.003)**	0.15 (0.667)	0.37 (0.103)
FD‐L	0.58 (0.061)	0.44 (0.046)	0.31 (0.357)	0.03 (0.892)
FD‐R	0.51 (0.111)	0.49 (0.026)	0.22 (0.511)	0.27 (0.230)
SUB‐L	0.52 (0.099)	**0.63 (0.002)**	0.34 (0.312)	0.19 (0.417)
SUB‐R	0.59 (0.058)	**0.55 (0.010)**	0.30 (0.369)	0.34 (0.129)
HATA‐L	0.41 (0.211)	0.27 (0.240)	0.20 (0.559)	0.56 (0.008)
HATA‐R	**0.79 (0.004)**	0.35 (0.120)	0.14 (0.690)	0.40 (0.071)

*Note:* Values are Pearson's *r* (exact *p*‐values in parentheses). *p < 0.05, **p ≤ 0.01 (uncorrected). False discovery rate correction (Benjamini–Hochberg, *q* = 0.05) was applied separately within each group × behavioral domain family (CB‐LoD, CB‐LoA, SC‐LoD, SC‐LoA) and within each hemisphere, correcting across the four hippocampal subfields (CA, FD, SUB, HATA). Correlations surviving FDR correction are indicated in bold.

Abbreviations: CB = congenitally blind; SC = sighted controls.

### 3.5 Obstacle Detection Learning Relationship With Hippocampal Subfield Volumes

LoD showed selective hippocampal volume–behavior relationships (Table 5; Figure 4). Correlations were computed separately within each group, and false discovery rate (FDR) correction was applied within each group × behavioral domain family (i.e., across hippocampal subfields for LoD in CB and SC independently).

In the CB group, FDR‐corrected analyses revealed significant positive associations between LoD and right CA as well as right HATA volumes. Prior to correction for multiple comparisons, additional positive correlations were observed between LoD and bilateral CA, FD, and SUB volumes (0.51 ≤ *r* ≤ 0.68), with the strongest uncorrected association involving right HATA (*r* = 0.79, *p* = 0.0036).

In sighted controls, LoD was significantly associated with bilateral CA and SUB volumes after FDR correction. Although additional subfields showed nominal positive correlations prior to correction, these did not survive FDR adjustment.

### 3.6 Obstacle Avoidance Learning Relationship With Hippocampal Subfield Volumes

In contrast to detection learning, LoA showed no significant associations with hippocampal subfield volumes in CB participants (Table 5). In sighted controls, LoA was positively associated with left HATA volume at the zero‐order level (*r* = 0.56, *p* = 0.008); however, this effect did not remain significant after controlling for total brain volume.

## 4 Discussion

Building on our prior ROI/PCA findings linking detection learning (LoD) to hippocampal volumes in sighted controls and to broader sensorimotor contributions in congenital blindness (Chebat et al. 2020a), the present work refines the anatomical specificity of these effects by localizing structure–learning relationships to distinct hippocampal subfields and hemispheres. Hippocampal subfield volumes showed the most robust associations with learning‐related measures, whereas complementary analyses of raw detection and avoidance performance during the training and test phases revealed weaker and less consistent relationships, particularly in the CB group.

This pattern suggests that hippocampal structure relates more consistently to experience‐dependent updating and relational learning than to moment‐to‐moment perceptual detection or motor execution. Congenital blindness was associated with selective subfield alterations and distinct subfield–behavior coupling, consistent with anatomically specific reorganization and experience‐dependent reweighting rather than uniform hippocampal involvement.

### 4.1 Subfield Specific Alterations of the Hippocampus in Congenital Blindness

Our findings clarify earlier reports of hippocampal structural differences in CB, which have yielded mixed results across studies (Bleau et al. 2022; Schinazi et al. 2016; Chebat et al. 2020b), some reporting reduced posterior hippocampal volume in CB (Chebat et al. 2007), while others described enlarged anterior hippocampal volumes associated with superior navigation performance (Fortin et al. 2008). These discrepancies may reflect differences in segmentation approaches and analytic scale, because global volumetry or coarse anterior–posterior divisions may obscure localized, subfield‐specific effects. Indeed, surface‐based morphometric analyses have reported spatially heterogeneous hippocampal alterations in CB and late blind individuals despite preserved total hippocampus proper volume (Pan et al. 2021).

Using a cytoarchitectonic subfield‐based approach, our results demonstrate that structural alterations in CB are selectively expressed, with the most robust effects localized to the left CA and, to a lesser extent, left FD. Other subfields exhibited consistent trend‐level reductions that did not reach statistical significance, suggesting a graded pattern of structural reorganization rather than uniform hippocampal scaling across subfields. This graded and subfield‐selective pattern is consistent with models of hippocampal regional specialization, in which different hippocampal territories support partially distinct computational demands (Poppenk et al. 2013; Strange et al. 2014). Thus, the present findings highlight subfield‐specific structural adaptations in CB rather than uniform reductions across the hippocampal formation (Figure 3 and Table 3).

Because blindness can affect overall brain size, hippocampal size, and internal hippocampal organization in partially independent ways, hippocampal subfield volumes were examined at two complementary levels. TBV‐adjusted volumes capture hippocampus‐specific effects beyond global brain scaling, whereas proportional analyses inform internal hippocampal organization. These analyses showed that CA occupies a smaller fraction of the hippocampus in CB, whereas FD, SUB, and HATA largely preserve their relative contributions despite reduced absolute volumes. Thus, CB appears to involve both overall hippocampal volume reduction and selective CA underrepresentation, providing an anatomical framework for interpreting the subfield‐specific structure–behavior relationships. CA subfields play a central role in allocentric spatial coding and relational memory (O'Keefe and Nadel 1978; Burgess et al. 2002; Moser et al. 2017; Leutgeb et al. 2007), functions that remain relevant even without vision. The selective involvement of CA is therefore anatomically plausible and consistent with evidence that sensory deprivation can differentially affect hippocampal subregions depending on their computational roles and network connectivity (Poppenk et al. 2013; Strange et al. 2014; Collin et al. 2015; O'Mara 2005; Aumont et al. 2023).

### 4.2 Hippocampal Subfield Contributions to Navigation Components

The present findings align with previous work showing that interindividual variability in brain structure within the hippocampus (Schinazi et al. 2013) and across non‐visual cortical systems in blindness (Voss and Zatorre 2012) predicts task‐specific performance and spatial learning (Chebat et al. 2007; Fortin et al. 2008; Leporé et al. 2009).

Taken together, these results indicate that hippocampal structure relates more strongly to experience‐dependent learning than to immediate perceptual detection or motor responses, consistent with the role of the hippocampus in encoding and refining spatial representations across experience (O'Keefe and Nadel 1978; Burgess et al. 2002; Moser et al. 2017; Poppenk et al. 2013; Strange et al. 2014; Collin et al. 2015), rather than mediating rapid sensorimotor reactions (Montello 2005; Wolbers and Hegarty 2010; Byrne et al. 2007; Yassa and Stark 2011; Leutgeb et al. 2007).

At the functional level, altered hippocampal network coupling along the longitudinal axis has been reported in congenitally and late blind individuals (Ma et al. 2017), suggesting large‐scale functional reorganization that may coexist with subfield‐specific structural contributions to navigation.

In the present data, learning of detection (LoD) was primarily associated with bilateral CA and SUB volumes in sighted controls and with right CA and HATA volumes in congenitally blind participants. This modest right‐lateralization is consistent with evidence linking right hippocampal circuitry to allocentric, map‐based spatial inference during navigation (Doeller et al. 2008). Given the role of CA in allocentric spatial coding and relational memory (O'Keefe and Nadel 1978; Burgess et al. 2002; Moser et al. 2017; Danjo 2020), the CA–LoD association likely reflects the need to infer obstacle layout from sequential sensory information across experience rather than relying solely on immediate contact‐based cues. This interpretation is consistent with psychological accounts of sensory‐substitution navigation as involving object identification, spatial localization, and action guidance (“what,” “where,” and “how”; Proulx et al. 2018), and with evidence that visual experience facilitates allocentric spatial representations (Pasqualotto et al. 2013). Associations involving other subfields did not survive correction for multiple comparisons, indicating selective rather than diffuse structure–behavior coupling, although small sample size may have reduced sensitivity to secondary effects. In SC, LoD was additionally associated with subicular volume, consistent with the subiculum's role in relaying hippocampal spatial representations to distributed cortical navigation networks (Leutgeb et al. 2007; O'Mara 2005). Although subicular associations did not survive FDR correction, trend‐level correlations suggest that subicular involvement may be present but less dominant. Instead, detection learning was selectively associated with right HATA volume. Because HATA lies at the interface between hippocampal memory circuits and amygdalar networks involved in salience processing and multisensory relevance (Pitkänen et al. 2000; Amunts et al. 2005), its involvement in CB may reflect greater reliance on salience‐based or integrative mechanisms when extracting spatial structure from non‐visual sensory signals (Kupers and Ptito 2014; Chebat et al. 2020b). Such a shift is compatible with experience‐dependent plasticity and preserved or enhanced spatial learning capacities following congenital visual deprivation (Pan and Monje 2020; Kupers and Ptito 2014; Tinti et al. 2006; Chebat et al. 2011, 2020a). In the present data, the strongest structure–learning relationships in CB involved subfields whose proportional representation within the hippocampus remained stable (Table 4), suggesting that learning‐related variability may be carried by subregions with relatively preserved internal representation despite overall volume loss.

These findings indicate that hippocampal volume–navigation relationships are subfield‐specific and that visual experience modulates how particular hippocampal subfields relate to performance. This interpretation aligns with broader evidence that experience‐dependent plasticity shapes behavior associations in blindness (Voss and Zatorre 2012; Kupers and Ptito 2014; Chebat et al. 2020a).

Although several subfields exhibited positive structure–learning slopes in both groups, fewer associations reached statistical significance in the CB group, likely reflecting the smaller CB sample size and reduced sensitivity to detect additional secondary effects in CB, which should be interpreted cautiously. However, the FDR‐surviving pattern was not identical across groups: bilateral CA and SUB volumes showed the most robust relationships with detection learning in SC, whereas right CA and right HATA predominated in CB.

### 4.3 Study Limitations

Several limitations should be considered when interpreting these findings. First, the CB sample was relatively small, reflecting the rarity of congenital blindness, which may have limited sensitivity to detect subtle group differences. Second, MRI data were acquired at 1.5 T, and hippocampal subfields were defined using atlas‐based probabilistic cytoarchitectonic ROIs. Although these methods provide anatomically validated and internally consistent subfield definitions, higher‐resolution acquisitions and dedicated medial temporal lobe imaging may allow finer characterization of hippocampal subregions in future studies. Third, the navigation task quantified learning as performance transfer between training and test phases rather than through trial‐by‐trial learning curves. While this approach was designed to capture generalization across spatial layouts, the retrospective dataset did not include trial‐level measures of TDU familiarization, response timing, or response lags, and the atlas‐based CA ROI did not allow CA1 to be isolated. Future studies using trial‐by‐trial behavioral measures, higher‐resolution medial temporal lobe imaging, and cytoarchitectonic or transcriptomic maps could further separate device‐specific learning from navigation‐specific learning. Nevertheless, complementary analyses of raw task performance yielded weaker and less consistent associations than those observed for learning measures, supporting the interpretation that the present findings relate primarily to navigation learning rather than static TDU‐mediated performance.

## 5 Conclusions

This study examined hippocampal subfield anatomy and its relationship to distinct components of navigation in congenitally blind and sighted adults. We show that CB is associated with selective, subfield‐specific structural differences and non‐uniform structure–behavior relationships, rather than global hippocampal alterations. CB is associated with reduced hippocampus proper volume, driven primarily by left CA differences, together with selective alterations in subfield composition. Structure–learning relationships also differed between groups. Detection learning was associated with bilateral CA and SUB volumes in sighted controls, whereas in congenitally blind participants, the strongest associations involved right CA and right HATA volumes. This group‐specific pattern indicates that early visual deprivation does not produce uniform hippocampal scaling but instead reflects anatomically specific reorganization and altered coupling between hippocampal subfield structure and spatial learning.

## Author Contributions

**Daniel‐Robert Chebat**, **Fabien C. Schneider**, **Ron Kupers** and **Maurice Ptito:** conceptualization. **Daniel‐Robert Chebat**, **Fabien C. Schneider**, **Ron Kupers** and **Maurice Ptito:** experimentation. **Daniel‐Robert Chebat:** methodology. **Fabien C. Schneider:** software. **Daniel‐Robert Chebat**, **Fabien C. Schneider**, and **Maurice Ptito:** validation. **Daniel‐Robert Chebat** and **Fabien C. Schneider:** formal analysis. **Daniel‐Robert Chebat:** investigation. **Maurice Ptito:** resources. **Fabien C. Schneider** and **Daniel‐Robert Chebat:** data curation. **Daniel‐Robert Chebat:** writing – original draft preparation. **Daniel‐Robert Chebat**, **Fabien C. Schneider**, **Ron Kupers**, and **Maurice Ptito:** writing – review and editing. **Daniel‐Robert Chebat:** visualization. **Maurice Ptito**, **Fabien C. Schneider**, and **Daniel‐Robert Chebat:** supervision. **Daniel‐Robert Chebat**, **Fabien C. Schneider**, and **Maurice Ptito:** project administration. **Maurice Ptito:** funding acquisition. All authors have read and agreed to the published version of the manuscript.

## Funding

This work was supported by the Harland Sanders Research Chair in Visual Science (University of Montreal; M.P.).

## Ethics Statement

The study was conducted in accordance with the Declaration of Helsinki. Ethical approval for the behavioral experiments was obtained from the relevant local ethics committees, including the Montreal Association for the Blind and the Centre for Interdisciplinary Research in Rehabilitation (CRIR), as well as the Institut Universitaire de Gériatrie de Montréal (IUGM). Ethical approval for the MRI component of the study was obtained through the appropriate institutional ethics committee affiliated with the Université de Montréal. The original approvals were granted prior to the routine inclusion of protocol codes in publications, and therefore approval numbers are not available.

## Consent

Informed consent was obtained from all subjects involved in the study.

## Conflicts of Interest

The authors declare no conflicts of interest.

## Data Availability

The data presented in this study are available from the corresponding author upon reasonable request. The data are not publicly available due to ethical and privacy restrictions.
